# Hydroalcoholic Extract of *Levisticum officinale* Increases cGMP Signaling Pathway by Down-Regulating *PDE5* Expression and Induction of Apoptosis in MCF-7 and MDA-MB-468 Breast Cancer Cell Lines

**DOI:** 10.29252/.23.4.280

**Published:** 2019-07

**Authors:** Marzieh Lotfian Sargazi, Ramin Saravani, Ali Shahraki

**Affiliations:** 1 Cellular and Molecular Research Center Zahedan University of Medical Sciences, Zahedan, Iran; 2 Department of Clinical Biochemistry, School of Medicine, Zahedan University of Medical Sciences, Zahedan, Iran; 3 Department of Biology, Faculty of Science, University of Sistan and Baluchestan, Zahedan, Iran

**Keywords:** Apoptosis, Breast cancer, Levisticum, *PDE5*

## Abstract

**Background:**

This study aimed to investigate *Levisticum officinale* hydroalcoholic extract (*LOHE*) effect on both cGMP signaling pathway and phosphodiesterase 5 (*PDE5*) gene expression pattern and to examine the role of LOHE in apoptosis induction of MCF-7 and MDA-MB-468 cell lines.

**Methods:**

The half maximal inhibitory concentration (IC50) of *LOHE* was examined in both cell lines using the MTT assay. Using IC50 values of *LOHE* on both cells, the type of cell death was detected by flowcytometric analysis. The values of *PDE5* and cGMP were evaluated by real-time PCR and ELISA methods, respectively.

**Results:**

The IC50 values were measured as 150 μg/ml for MDA-MB-468 and 200 μg/ml for MCF-7. At 12 hour of treatment, a significant decrease in the *PDE5* expression and maximum increase in the amount of intracellular cGMP were observed (*p* < 0.05). However, these effects were more noticeable in MDA-MB-468 triple-negative cells.

**Conclusion:**

Our data suggest that *LOHE* extract could be a potential source for new strategies towards targeting both *PDE5* and cGMP signaling pathways.

## INTRODUCTION

Breast cancer is a type of malignancy originating from breast tissue and may spread to other vital organs such as bones, lung, liver, and brain[1]. This cancer is one of the most common malignancies in women and the second leading cause of cancer-related death among females worldwide[2]. Based on world statistics, there is an increasing incidence of breast cancer, especially in developing countries where the lower rates of this sex-related tumor have been reported[3]. Despite all efforts to prevent or treat, the disease has remained a major global health problem[4].

Exploration of novel markers for early diagnosis and therapeutic targets, which are directly involved in cancerous cell pathways, can be considered as a major challenge in cancer research[5,6]. A novel cancer-related marker is phosphodiesterase (PDE) enzyme. The PDE enzyme family members are in fact metallohydrolase proteins that break down the phosphodiester bond of the cyclic nucleotides cyclic adenosine monophosphate (cAMP) and/or cyclic guanosine monophosphate (cGMP) into the inactive 5’-AMP or GMP[7,8]. *PDE5* mechanism of action is specific for hydrolysis of cGMP. PDE5 is also the predominant isoform that hydrolyzes cGMP in almost all tissues, which later causes cGMP signaling pathway to be terminated as long as intracellular cGMP levels are controlled by negative feedback regulation[8-10]. cGMP can activate cGMP-dependent protein kinase (PKG) and PDE enzymes. It also causes ion fluxes and protein phosphorylation that can affect genes expression or other cellular responses[6,11,12]. Recently, the overexpression of *PDE5* has been reported in several human carcinomas, including breast cancer, and suggested that *PDE5* expression has positive correlation with tumor grade, lymph node involvement, and invasive potential, as well as decreasing survival rate in patients. Additionally, cGMP amount and PKG seem to have negative association with each other[5,12-15].

*PDE5* is differentially expressed in every subtype of breast cancer cells, including luminal A (ER-positive/PR + HER2 negative), luminal B (ER + and/or PR + HER2+), HER2-enriched (ER- and PR-/HER2+), and triple-negative (i.e., the lack of all three receptors). Luminal A subtype, like the MCF-7 cell line, is the most frequent breast cancer subtype that is low grade, tends to grow slowly and has the highest survival rate, whereas triple-negative, similar to MDA-MB-468, is less common breast cancer subtype, has the lowest survival rate, has very high aggressive potentials and is difficult to treat because no hormone receptor has been found to target yet. This subtype expresses higher levels of *PDE5* compared to other subtypes[16,17].

Several studies have suggested that *PDE5* and cGMP signaling pathways can be considered as new candidates for discovering novel therapeutic strategies toward treating breast cancer, especially triple-negative subtype[12,18,19]. Nevertheless, common inhibitors of *PDE5* have been reported to possess a well-established side effect in clinical experiments. Extracts of plants or their bioactive compounds are found to be safe and are widely acceptable in cancer therapy[20-22]. Thus, herbal plants are essentially a potential source for developing novel drugs in the treatment of cancer[23-25]. Studies have shown that some plant extracts have anticancer activities by inhibiting proliferation and inducing cell cycle arrest beside suppressing tumor progression *in vitro* and *in vivo*[26,27].

*Levisticum officinale* (lovage) is regarded as a herbaceous perennial herb of the family Umbelliferae (Apiaceae). It is a wild herb that grows in various areas of Europe, Afghanistan, and Iran (i.e. Kerman Province). According to previous investigations, lovage is used to treat sore throats, fever, kidney stones, urethritis, congestion, rheumatism, migraine headache, and indigestion and also applied as a wound antiseptic. Lovage has also used as an appetizer, and a potent diuretic[28-30] and has anti-inflammatory, antioxidant, anti-tumoral and anti-bacterial properties[28,31-33]. Experiments have revealed that hydroalcoholic extract of lovage (*LOHE)* has anti-cancer effects by induction of apoptosis in several cancer cell lines[32,34,35], based on the fact that plants containing flavonoids can cause PDE inhibition. The aim of the present study was to investigate the effect of *LOHE* on both *PDE5* expression and cGMP signaling pathway to evaluate the role of *LOHE* in apoptosis induction in both breast cancer cell lines, MCF-7 (ER+, PR+, HER2-), and MDA-MB-468 (triple-negative).

## MATERIALS AND METHODS

### Chemicals and reagents

RPMI 1640 culture medium, FBS, PBS, penicillin, streptomycin, and Trypsin/EDTA solution were all purchased from Gibco (Rockville, MD, USA). MTT, Trypan blue, and dimethyl sulfoxide (DMSO) were procured from Sigma Aldrich (St. Louis, MO, USA). The Annexin V/PI apoptosis detection kit was obtained from BioVision (San Francisco, CA, USA). The RevertAid M-MuLV Reverse Transcriptase and the cGMP Direct Immunoassay kit were obtained from Takara Bio Inc. (Dalian, China) and R&D Systems (Minneapolis, MN, USA), respectively. All other materials were of analytical grade.

### Plant materials

*L. officinale* (lovage) was collected in spring time from a small area in Southeast of Iran, Hezar Mountains, ranging from 3000 to 3400 meters from the sea level. Toxonomy of Lovage was confirmed by Department of Biology, University of Sistan and Baluchistan, Zahedan, Iran.

### Preparation of hydroalcoholic extract

Extract of 70% water-alcohol was prepared with the Soxhlet extractor. The aerial part of the plant was dried in the dark at room temperature. Then 20 g of the dried plant stems and leaves were added to 300 ml of alcohol 70% and placed in the Soxhlet device. Afterwards, the extract was filtered (Whatman No. 41), and the solvent was removed using a freeze dryer machine (MAXI DRY-LYO, Heto-Holten, Allerod, Denmark). Primary Stock made from 100 mg of *LOHE* was dissolved in 1 ml of DMSO (HPLC grade) and kept in -20 °C for further use.

### Cell culturing

Human breast cancer cell lines, MCF-7 and MDA-Mb-468, were purchased from the National Cell Bank of Pasteur Institute of Iran (Tehran). The cells were cultivated in RPMI 1640 medium containing 10% FBS, 100 U/ml of penicillin, and 100 μg/mL of streptomycin under standard cell culture conditions (95% humidity, 37 °C, 5% CO_2_). The culture medium within the flask was replaced with a fresh medium every 2-3 days. Prior to each assay, cells were counted, and the number of living cells were calculated with Hemocytometer and by Trypan blue assay. All assays were done at least in triplicates.

### Cell viability

MTT assay was used to evaluate cytotoxicity. Almost 5000 cells per well were seeded in 96-well microplates and let to grow until the confluency of 80%. The culture medium was removed, and cells were treated with the concentrations of 0 (DMSO containing culture medium), 50, 100, 150, 200, 300, 400, and 500 μg/mL of *LOHE* and incubated for 24, 48, and 72 hours. Then 20 μL of MTT solution was added to treated and untreated cells. Following 4 h of incubation at 37 °C, the solution within microwells was carefully drained, and 150 μL of DMSO was added to each microwell and kept in the dark for 20 min. Absorptions were read at 570 nm using a microplate reader (Stat Fax 2100; Awareness Technology, Los Angeles, CA, USA), and cell viability was measured.

### Apoptosis assay

Apoptotic induction rate in cells treated with different concentrations of the extract, and untreated cells was evaluated using Annexin V-FITC apoptosis detection kit (BioVision) according to the manufacturer’s protocol. Briefly, 1 × 10^5^ cells per well were seeded in six-well plates. Then the cells were treated with increasing doses of *LOHE*, 0, 50, 100, 150, 200 μg/ml for MDA-Mb-468 and 0, 100, 200, 300 μg/ml for MCF-7. Afterwards, the medium was discarded, and the cell pellet was transferred to a microtube before centrifuging at 12000 rpm for 10 minutes. Later, 500 μL of 1× binding buffer and 5 μL of Annexin V-FITC were added to cell suspension and incubated in the darkness at room temperature for 10 minutes. Finally, 5 μL of propidium iodide was added to cell pellets, and the samples were analyzed by using a Pas-II cytometer (Partec AG, CH-4144 Arlesheim, Switzerland).

### Extraction of total RNA and synthesis of complementary DNA (cDNA)

Cells were cultured in six plates (1 × 10^5^ cells per well) and were treated with the concentration equal to IC50 values of *LOHE* for both cells (150 μg/ml for MDA-MB-468 and 200 μg/ml for MCF-7) at 2, 4, 8, 12, and 24 h after treatment. Total RNA was isolated using RNX (SinaClon, Tehran, Iran) according to the manufacturer’s instruction. cDNA was synthesized using the Takara first strand cDNA synthesis kit (Dalian, China) based on the protocol provided by manufacturer.

### Real-time polymerase chain reaction (PCR) assay

Real-time PCR was performed for relative mRNA expression of *PDE5*. The *PDE5* primers were designed, ensuring that all the isoforms of *PDE5* were amplified. *PDE5* and *GAPDH* primer sequences were as follows: 5’-TGTTGGTGTAGCACAGACCA-3’ and 5’-GAGCCACATCGCTCAGACAC-3’ as forward and 5’-GCAGTGAAGTCTGATAGAGC-3’ and5’-CATGT AGTTGAGGTCAATGAAGG-3’ as reverse primers, respectively. PCR amplification consisted of 35 cycles: 95 °C for 15 seconds, 58.5 °C for 30 seconds, and 72 °C for 45 seconds. The housekeeping gene, GAPDH, was used to normalize the relative expression level of the *PDE5* gene. Relative expression of *PDE5* was compared with *GAPDH* (as the internal control) using the comparative 2^−ΔΔct^ method.

### Measurement of cGMP concentration

cGMP levels in both cells were measured by ELISA method using the cGMP direct immunoassay kit (R&D Systems, Minneapolis, MN, USA). Briefly, in this assay, cGMP present in a sample competes with a constant amount of peroxidase-conjugated cGMP for sites on rabbit polyclonal antibody during the incubation. Hence, the polyclonal antibody binds the pre-coated goat anti-rabbit antibody. Eventually, cGMP levels were measured based on absorptions at 450 nm.

### Statistical analysis

SPSS software version 22 (SPSS Inc., Chicago, IL, USA) was used for the statistical analysis of the data, and the findings were reported as mean ± SD. The statistical analysis was conducted by a nonparametric analysis of variance between the groups. *p* < 0.05 was considered statistically significant in all assays.

## RESULTS

### Antiproliferative effects of LOHE

**Fig. 1:**
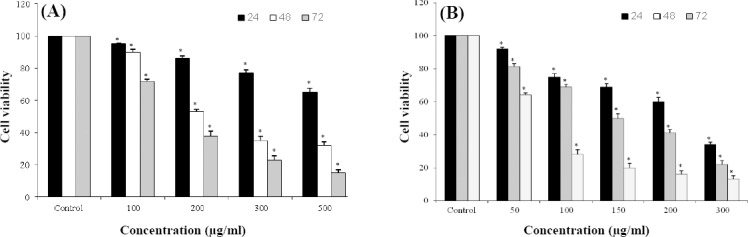
Concentration-response analysis of *LOHE* on both (A) MCF-7 and (B) MDA-MB-468 cells (^*^p < 0.05).

*LOHE* significantly reduced viability in both MCF-7 (Fig. 1A) and MDA-MB-468 (Fig. 1B) cell lines in a concentration- and time-dependent manner (*p* < 0.05). The most significant inhibitory effect of *LOHE* was at 300 μg/ml and 500 μg/ml after 72 h of treatment in MDA-MB-468 and MCF-7 cell lines, respectively. Moreover, the results showed that the IC50 values of *LOHE* were 150 μg/ml for MDA-MB-468 cells and 200 μg/ml for MCF-7, following 48 h of treatment.

### Induction of apoptosis by LOHE

**Fig. 2:**
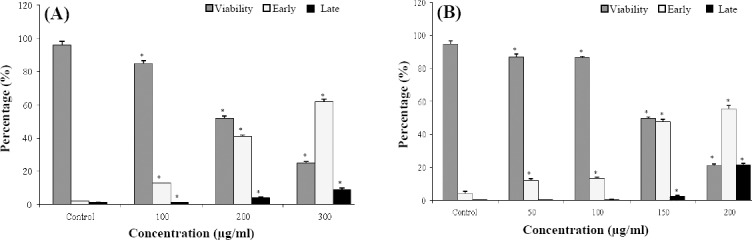
Flowcytometric analysis of death cell types after 48 h exposure to different concentrations of *LOHE* in (A) MCF-7 and (B) MDA-MB-468 cell lines (^*^p < 0.05).

To investigate the apoptosis inducing potency of *LOHE*, MCF-7 and MDA-MB-468 cell lines were treated with different concentrations of this extract and incubated for 48 h. As shown in Figure 2, there were significant increases in the early and the late apoptosis rate of both cells in a concentration-dependent manner (*p* < 0.05), but the fraction of MDA-MB-468 cells undergoing apoptosis was higher compared to MCF-7 cells treated with the same concentrations of *LOHE*.

### Effect of LOHE on PDE5 mRNA gene expression

**Fig. 3:**
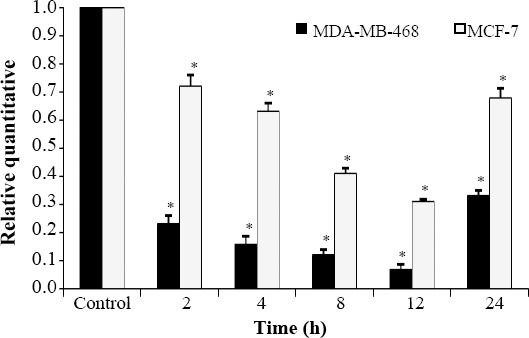
*PDE5* mRNA expression assay in ER+ and ER- cell lines of human breast cancer cell lines, MCF-7 and MDA-MB-468 following treatment with *LOHE* (^*^p < 0.05).

Effect of *LOHE* on *PDE5* expression was assessed in a time-dependent manner in both MCF-7 and MDA-MB-468 cell lines. As shown in Figure 3, the *PDE5* mRNA levels significantly reduced in MCF-7 cells in the presence of *LOHE* over treatment periods of 2, 4, 8, 12, and 24 h compared to that of the untreated groups (*p* < 0.05). A similar effect was found in MDA-MB-468 cells as well. However, the inhibitory effect of *LOHE* on MDA-MB-468 was more significant than MCF-7 cells in all the measured periods (*p* < 0.05).

### cGMP intracellular assay

**Fig. 4:**
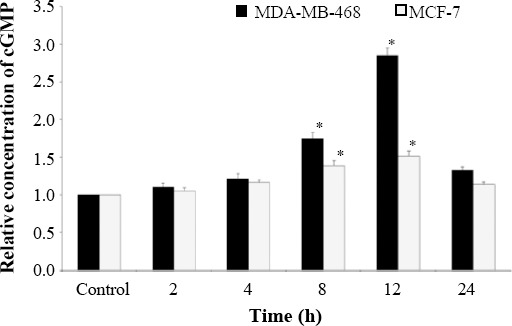
Effects of *LOHE* on intracellular cGMP levels in both MCF-7 and MDA-MB-468 cell lines (^*^p < 0.05) .

As shown in Figure 4, *LOHE* significantly increased cGMP concentrations at 8-12 h exposure periods in both MCF-7 and MDA-MB-468 cell lines compared to untreated cell lines (*p* < 0.05). The highest cGMP levels in MCF-7 and MDA-MB-468 cell lines were at 12 h treatment. Results indicated that intracellular cGMP levels increased in both cell lines following treatment with *LOHE* compared to the adjacent untreated controls (*p* < 0.05). As a result, promoted level of cGMP suggests the presence of a major regulator of basal cGMP levels in both cell lines.

## DISCUSSION

The cGMP signaling plays an important role in cell proliferation, differentiation, angiogenesis, apoptosis, and tumor-logical activity[18]. The mRNAs of specific-cGMP PDEs such as *PDE5* have been shown to be increased in various cancers; thus, the inhibitors of this enzyme could be suitable therapeutic candidates for breast cancer[19]. Previously, we have demonstrated that PDE9 inhibition and activation of cGMP signaling are substantially associated with the breast cancer cell growth inhibitory effects of BAY 73-6691, an apoptosis-inducing factor[36]. The cGMP-specific PDEs include *PDE5*, *6*, and *9*[37-40].

Recently, a tendency to drugs of plant origin has been raised. Herbal medicines are extremely valuable compounds for discovery of novel PDE5 inhibitors[41]. Essential oil of Lovage significantly regulates p53 pathways, which is an important tumor suppressor pathways[32]. In a study conducted by Sertel *et al*.[32], essential oil extract of lovage showed an IC50 value of 292.6 μg against USCC1 head and neck squamous carcinoma cells using the XTT method. Another study by Bogucka-Kocka and colloquies[34] on the effect of lovage hydroalcoholic extract on nine cell lines of human leukemia demonstrated IC50 values of 240 μg/ml for 1301 cell lines, 187 μg/ml for ML-1, 150 μg/ml for Eol, 135 μg/ml for H-9, 300 μg/ml for HL-60, 28 μg/ml for J45, 225 μg/ml for U266, 67 μg/ml for WICL, and 24 μg/ml for C8166 using Trypan blue assay. Well-defined signaling pathways have recently been proposed for the role of this enzyme in cancerous cell. In fact, the high expression of *PDE5* in cancers causes a reduction in cGMP through hydrolysis, hence decreasing PKG activity. PKG increases β-catenin phosphorylation and decreases β-catenin expression and protein levels; thus, PKG with the inhibition of β-catenin/T cell factor/lymphoid enhancer factor promotes the down-regulation of apoptosis-inhibiting proteins such as cyclin D1[15,42]. Also, Wnt/β-catenin pathway can be activated by Rho, the family of GTP-binding proteins. *PDE5* overexpression increased motile and invasive properties of cells through the activation of the Rho family. These proteins affect cell migration and metastasis. Besides, a cGMP-PKG cascade can inhibit Rho in different cell types[43,44]. *PDE5* up-regulation is essential in cancer progression for the reason that cGMP signaling may be independent of other common breast cancer signaling pathways (hormone receptors or HER-2). This pathway can suppress proliferation and induce apoptosis in breast cancer. Studies have shown a negative correlation between cGMP/PKG and Wnt-β-catenin pathways, associating with a wide range of genes and proteins involved in the tumorigenesis[5,15,42]. Dysregulation of Wnt signaling plays a crucial role in the development and progression of triple-negative breast cancer[45,46]. It has been reported that flavonoids, especially quercetin and coumarin, are able to inhibit potential Wnt/β-catenin signaling[47,48].

Regarding the effects of *LOHE* on *PDE5* mRNA levels, there were significant differences between MDA-MB-468 and MCF-7 cells at the times of 2-24 h, particularly at 12-h in this period. The mRNA level re-increased after 24 h in both cell lines, even though the elevation in *PDE5* mRNA in MDA-MB-468 cell line was more evident than that of MCF-7 cell line. Our results showed that *LOHE* could increase significantly the intracellular cGMP levels in both cell lines. This feature of *LOHE* was correlated with adjacent *PDE5* expression alterations and intracellular cGMP levels. As an example, during 8-12 h, *PDE5* expression was minimum, and consequently, cGMP levels were maximum. However, the increased amounts of cGMP showed anti-proliferative effects of *LOHE* in both cell lines. This increase was observed more in triple-negative MDA-MB-468 cells compared to MCF-7 cells.

In summary, our findings show that *LOHE* inhibits proliferation and induces apoptosis in two cell lines. In addition, *LOHE* could be a novel source of drug candidates in breast cancer treatment, especially for those types of cancer, which there are fewer treatment options and limited markers for pharmaceutical target (no hormone receptor). The high expression of *PDE5* can be a new marker for this subtype, and inhibition of this enzyme with compounds derived from this plant may be pursued in clinic in near future.

## References

[1] Jin X, Mu P (2015). Targeting breast cancer metastasis. Breast cancer:basic and clinical research.

[2] Kazarian A, Blyuss O, Metodieva G, Gentry-Maharaj A, Ryan A, Kiseleva EM, Prytomanova OM, Jacobs IJ, Widschwendter M, Menon U, Timms JF (2017). Testing breast cancer serum biomarkers for early detection and prognosis in pre-diagnosis samples. British journal of cancer.

[3] Shulman LN, Willett W, Sievers A, Knaul FM (2010). Breast cancer in developing countries:opportunities for improved survival. Journal of oncology.

[4] Lukong KE (2017). Understanding breast cancer–The long and winding road. BBA clinical.

[5] Catalano S, Campana A, Giordano C, Győrffy B, Tarallo R, Rinaldi A, Bruno G, Ferraro A, Romeo F, Lanzino M, Naro F, Bonofiglio D, Andò S, Barone I (2016). Expression and function of phosphodiesterase type 5 in human breast cancer cell lines and tissues:implications for targeted therapy. Clinical cancer research.

[6] Tinsley HN, Gary BD, Keeton AB, Zhang W, Abadi AH, Reynolds RC, Piazza GA (2009). Sulindac sulfide selectively inhibits growth and induces apoptosis of human breast tumor cells by phosphodiesterase 5 inhibition, elevation of cyclic GMP, and activation of protein kinase G. Molecular cancer therapeutics.

[7] Ahmad F, Murata T, Simizu K, Degerman E, Maurice D, Manganiello V (2015). Cyclic nucleotide phospho-diesterases:important signaling modulators and therapeutic targets. Oral diseases.

[8] Fajardo AM, Piazza GA, Tinsley HN (2014). The role of cyclic nucleotide signaling pathways in cancer:targets for prevention and treatment. Cancers (Basel).

[9] Corinaldesi C, Di Luigi L, Lenzi A, Crescioli C (2016). Phosphodiesterase type 5 inhibitors:back and forward from cardiac indications. Journal of endocrinological investigation.

[10] Tinsley HN, Gary BD, Thaiparambil J, Li N, Lu W, Li Y, Maxuitenko YY, Keeton AB, Piazza GA (2010). Colon Tumor Cell Growth-inhibitory activity of sulindac sulfide and other nonsteroidal anti-inflammatory drugs is associated with phosphodiesterase 5 inhibition. Cancer prevention research (Phil).

[11] Tuttle TR, Mierzwa ML, Wells SI, Fox SR, Ben-Jonathan N (2016). The cyclic GMP/protein kinase G pathway as a therapeutic target in head and neck squamous cell carcinoma. Cancer letters.

[12] Windham PF, Tinsley HN (2015). cGMP signaling as a target for the prevention and treatment of breast cancer. Seminars in cancer biology.

[13] Peak TC, Richman A, Gur S, Yafi FA, Hellstrom WJ (2016). The role of PDE5 inhibitors and the NO/cGMP pathway in cancer. Sexual medicine reviews.

[14] Fallahian F, Karami-Tehrani F, Salami S, Aghaei M (2011). Cyclic GMP induced apoptosis via protein kinase G in oestrogen receptor-positive and-negative breast cancer cell lines. The FEBS journal.

[15] Tinsley HN, Gary BD, Keeton AB, Lu W, Li Y, Piazza GA (2011). Inhibition of PDE5 by Sulindac sulfide selectively induces apoptosis and attenuates oncogenic Wnt/β-catenin–mediated transcription in human breast tumor cells. Cancer prevention research (Phila).

[16] Fallahpour S, Navaneelan T, De P, Borgo A (2017). Breast cancer survival by molecular subtype:a population-based analysis of cancer registry data. CMAJ open.

[17] Bianchini G, Balko JM, Mayer IA, Sanders ME, Gianni L (2016). Triple-negative breast cancer:challenges and opportunities of a heterogeneous disease. Nature reviews clinical oncology.

[18] Barone I, Giordano C, Bonofiglio D, Catalano S, Andò S (2015). Phosphodiesterase type 5 as a candidate therapeutic target in cancers. Current pathobiology reports.

[19] Das A, Durrant D, Salloum FN, Xi L, Kukreja RC (2015). PDE5 inhibitors as therapeutics for heart disease, diabetes and cancer. Pharmacology and therapeutics.

[20] Kumar A, Sharma V, Singh VP, Kaundal M, Gupta MK, Bariwal J, Deshmukh R (2015). Herbs to curb cyclic nucleotide phosphodiesterase and their potential role in Alzheimer's disease. Mechanisms of ageing and development.

[21] Huang SA, Lie JD (2013). Phosphodiesterase-5 (PDE_5_) inhibitors in the management of erectile dysfunction. Pharmacy and therapeutics.

[22] Balhara YPS, Sarkar S, Gupta R (2015). Phosphodiesterase-5 inhibitors for erectile dysfunction in patients with diabetes mellitus:A systematic review and meta-analysis of randomized controlled trials. Indian journal of endocrinology and metabolism.

[23] Sandhya T, Mishra K (2006). Cytotoxic response of breast cancer cell lines, MCF 7 and T 47 D to triphala and its modification by antioxidants. Cancer letters.

[24] Bayala B, Bassole IH, Gnoula C, Nebie R, Yonli A, Morel L, Figueredo G, Nikiema JB, Lobaccaro JM, Simpore J (2014). Chemical composition, antioxidant, anti-inflammatory and anti-proliferative activities of essential oils of plants from Burkina Faso. PLoS one.

[25] Newman DJ, Cragg GM, Snader KM (2003). Natural products as sources of new drugs over the period 1981-2002. Journal of natural products.

[26] Shaikh R, Pund M, Dawane A, Iliyas S (2014). Evaluation of anticancer, antioxidant, and possible anti-inflammatory properties of selected medicinal plants used in Indian traditional medication. Journal of traditional and complementary medicine.

[27] Millimouno FM, Dong J, Yang L, Li J, Li X (2014). Targeting apoptosis pathways in cancer and perspectives with natural compounds from mother nature. Cancer prevention research (Phila).

[28] Raal A, Arak E, Orav A, Kailas T, Müürisepp M (2008). Composition of the essential oil of *Levisticum officinale* W.D.J. Koch from some European countries. Journal of essential oil research.

[29] Khodashenas M, Keramat B, Emamipoor Y (2015). Callus induction and PLBs production from *Levisticum officinale* koch (a wild medicinal plant). Journal of applied environmental and biological sciences.

[30] Verdian Rizi MR, Abbas H (2007). The essential oil composition of *Levisticum officinalis* from Iran. Asian journal of biochemistry.

[31] Schinkovitz A, Stavri M, Gibbons S, Bucar F (2008). Antimycobacterial polyacetylenes from *Levisticum officinale*. Phytotherapy research.

[32] Sertel S, Eichhorn T, Plinkert PK, Efferth T (2011). Chemical Composition and antiproliferative activity of essential oil from the leaves of a medicinal herb *Levisticum officinale* against UMSCC1 head and neck squamous carcinoma cells. Anticancer research.

[33] Moradalizadeh M, Akhgar M, Rajaei P, Faghihi-Zarandi A (2012). Chemical composition of the essential oils of *Levisticum officinale* growing wild in Iran. Chemistry of natural compounds.

[34] Bogucka-Kocka A, Smolarz H, Kocki J (2008). Apoptotic activities of ethanol extracts from some Apiaceae on human leukaemia cell lines. Fitoterapia.

[35] El-Hamid SA, Abeer Y, Hendawy S (2009). Anti-inflammatory, antioxidant, anti-tumor and physiological studies on *Levisticum officinale*-Koch plant. Planta medica.

[36] Saravani R, Karami-Tehrani F, Hashemi M, Aghaei M, Edalat R (2012). Inhibition of phosphodiestrase 9 induces cGMP accumulation and apoptosis in human breast cancer cell lines, MCF-7 and MDA-MB-468. Cell proliferation.

[37] Hogg CL, Svoboda KP, Hampson JB, Brocklehurst S (2001). Investigation into the composition and bioactivity of essential oil from lovage (*Levisticum officinale* WDJ Koch). International journal of aromatherapy.

[38] Venskutonis PR (2016). Lovage (*Levisticum officinale* Koch.). Oils. Essential Oils in Food Preservation, Flavor and Safety.

[39] Najda A, Wolski T, Dyduch J, Baj T (2003). Determination of quantitative composition of poliphenolic compounds occur in anatomically different parts of *Levisticum officinale* Koch. Electronic journal of Polish agricultural universities, series horticulture.

[40] Wojdyło A, Oszmiański J, Czemerys R (2007). Antioxidant activity and phenolic compounds in 32 selected herbs. Food chemistry.

[41] Rahimi R, Ghiasi S, Azimi H, Fakhari S, Abdollahi M (2010). A review of the herbal phosphodiesterase inhibitors;future perspective of new drugs. Cytokine.

[42] Lee K, Piazza AG (2017). The interaction between the Wnt/β-catenin signaling cascade and PKG activation in cancer. Journal of biomedical research.

[43] Sauzeau V, Rolli-Derkinderen M, Marionneau C, Loirand G, Pacaud P (2003). RhoA expression is controlled by nitric oxide through cGMP-dependent protein kinase activation. Journal of biological chemistry.

[44] Morelli A, Filippi S, Sandner P, Fibbi B, Chavalmane AK, Silvestrini E, Sarchielli E, Vignozzi L, Gacci M, Carini M, Vannelli GB, Maggi M (2009). Vardenafil modulates bladder contractility through cGMP-mediated inhibition of RhoA/Rho kinase signaling pathway in spontaneously hypertensive rats. The journal of sexual medicine.

[45] Ji J, Zhang Y, Redon CE, Reinhold WC, Chen AP, Fogli LK, Holbeck SL, Parchment RE, Hollingshead M, Tomaszewski JE, Dudon Q, Pommier Y, Doroshow JH, Bonner WM (2017). Phosphorylated fraction of H2AX as a measurement for DNA damage in cancer cells and potential applications of a novel assay. PloS one.

[46] Pohl SG, Brook N, Agostino M, Arfuso F, Kumar AP, Dharmarajan A (2017). Wnt signaling in triple-negative breast cancer. Oncogenesis.

[47] Baruah MM, Khandwekar AP, Sharma N (2016). Quercetin modulates Wnt signaling components in prostate cancer cell line by inhibiting cell viability, migration, and metastases. Tumor biology.

[48] Amado NG, Fonseca BF, Cerqueira DM, Neto VM, Abreu JG (2011). Flavonoids:potential Wnt/beta-catenin signaling modulators in cancer. Life sciences.

